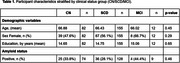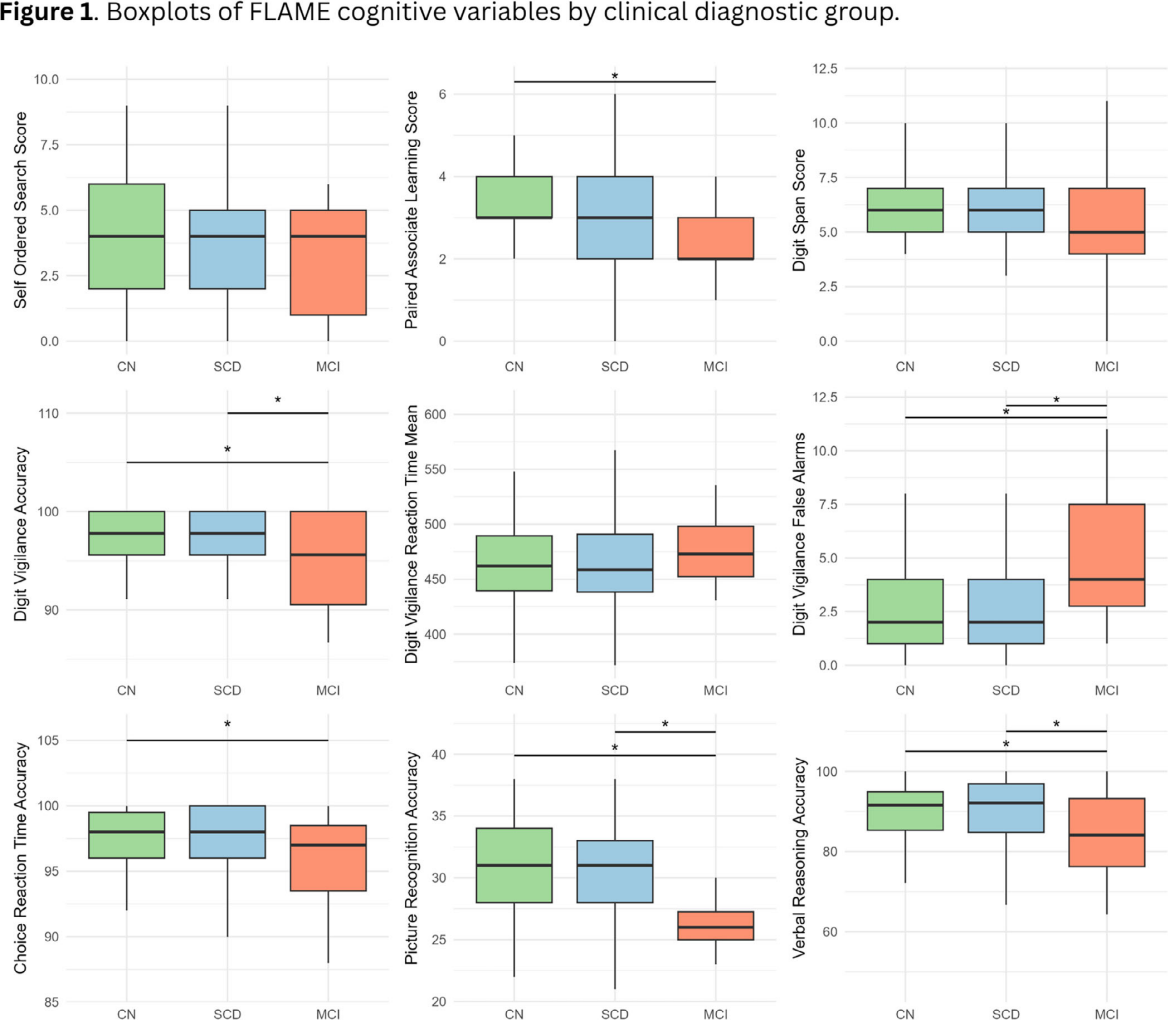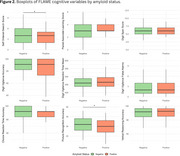# An unsupervised remote cognitive assessment predicts mild cognitive impairment and associates to amyloid status

**DOI:** 10.1002/alz70861_108840

**Published:** 2025-12-23

**Authors:** Clàudia Porta‐Mas, Anna Brugulat‐Serrat, Anne Corbett, Marc Suárez‐Calvet, Juan Domingo Gispert, Gemma Salvadó, Oriol Grau‐Rivera, Gonzalo Sánchez‐Benavides

**Affiliations:** ^1^ Barcelonaβeta Brain Research Center (BBRC), Pasqual Maragall Foundation, Barcelona Spain; ^2^ Hospital del Mar Research Institute (IMIM), Barcelona Spain; ^3^ College of Medicine and Health, University of Exeter, Exeter UK; ^4^ Servei de Neurologia, Hospital del Mar, Barcelona Spain; ^5^ Centro de Investigación Biomédica en Red de Fragilidad y Envejecimiento Saludable (CIBERFES), Instituto de Salud Carlos III, Madrid Spain; ^6^ AstraZeneca, Barcelona Spain; ^7^ Department of Clinical Sciences, Clinical Memory Research Unit, Lund University, Lund Spain

## Abstract

**Background:**

The use of digital biomarkers to assess cognition in Alzheimer’s disease (AD) offers scalable, efficient alternatives to paper‐and‐pencil tests. Validating these tools against clinical and biomarker‐defined groups is critical for their adoption in research, clinical trials and clinical contexts. This study evaluates the performance of a remote, unsupervised cognitive assessment (FLAME‐Factors of Longitudinal Attention, Memory and Executive Function) in distinguishing cognitive profiles across diagnostic categories and amyloid status in two cohorts from BBRC.

**Method:**

Cognitively normal (CN) participants from ALFA+ cohort and subjective cognitive decline (SCD) or mild cognitive impairment (MCI) patients from Beta‐AARC cohort were invited via email to FLAME remote and unsupervised assessment. 249 participants completed FLAME tasks, that include working memory (Self Ordered Search Score, Paired Associate Learning Score, Digit Span Score), episodic memory (Picture Recognition Accuracy), attention (Digit Vigilance Accuracy, Digit Vigilance False Alarms, Digit Vigilance Reaction Time Mean, Choice Reaction Time Accuracy) and executive function (Verbal Reasoning Accuracy). Analysis of covariance (ANCOVA) with post‐hoc (Tukey) were used to examine differences by clinical and amyloid status. Logistic regression models were employed to evaluate if the digital tasks predicted MCI. All analyses were adjusted for age, sex, and education.

**Result:**

MCI group showed reduced performance in paired associate learning score, attention variables, picture recognition accuracy and verbal reasoning compared to CN and SCD participants. Digit vigilance false alarms, picture recognition accuracy and verbal reasoning accuracy were able to significantly distinguish between CN and SCD groups (Figure 1).

Several cognitive variables significantly predicted MCI, including paired associate learning score (OR=1.93,95%CI[1.09‐3.51],*p*=0.02), digit vigilance accuracy (OR=1.17,95%CI[1.02‐1.35],*p*=0.02) and false alarms (OR=1.4,95%CI[1.13‐1.76],*p*=0.002), and accuracy from choice reaction time task (OR=1.23,95%CI[1.03‐1.47],*p*=0.01), picture recognition (OR=1.39,95%CI[1.17‐1.72],*p*<0.001), and verbal reasoning (OR=1.05,95%CI [1.01‐1.11],*p*=0.03).

Additionally, self ordered search score and picture recognition accuracy were significantly lower in amyloid‐positive individuals (Figure 2).

**Conclusion:**

A remote unsupervised assessment reliably differentiates diagnostic and AD biomarker‐defined groups and predicts MCI, underscoring its promise value for research and clinical contexts.